# All-arthroscopic repair of Atzei class II and III triangular fibrocartilage complex tears using the FasT-Fix suture device

**DOI:** 10.1186/s13018-020-02046-1

**Published:** 2021-03-24

**Authors:** Mengchun Tsai, Yi-Hsuan Lin, Chih-Hao Chiu, Chun-Ying Cheng, Yi-Sheng Chan, Alvin Chao-Yu Chen

**Affiliations:** 1 Bone and Joint Research Center, Department of Orthopaedic Surgery, Chang Gung Memorial Hospital–Linkou, 5, Fu-Shin Street, Kweishan District, Taoyuan, 333 Taiwan, Republic of China; 2 College of Medicine, Chang Gung University, Taoyuan, Taiwan, Republic of China

**Keywords:** Triangular fibrocartilage complex, Distal radioulnar joint, Peripheral tear, Arthroscopy

## Abstract

**Background:**

The study is aimed to propose an arthroscopic repair technique using a pre-tied suture device for peripheral TFCC (triangular fibrocartilage complex) tear with proximal component involvement.

**Methods:**

Through a retrospective review in the medical records of patients who underwent unilateral arthroscopic repair for TFCC Palmer IB lesion between 2017 and 2019, 12 patients were arthroscopically diagnosed as proximal component tear and received more than 1 year follow-up postoperatively. The arthroscope was introduced from 6R portal to discriminate Atzei class II from III lesions by a “visualization test” and to supervise the repair procedure using pre-tied FasT-Fix suture device from 3-4 portal. Two poly-ether-ether-ketone (PEEK) blocks were further advanced along the device needle to finally seat outside the ulnar joint capsule, followed by gradually tightening the pre-tied suture loop until the TFCC periphery was securely repositioned and held stably.

**Results:**

Operation time averaged 87 min. Hook test and DRUJ arthroscopy confirmed proximal component tear in all 12 wrists. Four patients were diagnosed to be Atzei class II lesion as full thickness tear of distal component was arthroscopically identified from 6R portal while the other 8 exhibited partial thickness tear and were categorized as class III lesion. Follow-up averaged 15 months with a range of 12 to 24 months. Mayo modified wrist score improved from an average of 61.3 preoperatively to 90.4 at the latest visit.

**Conclusions:**

A modified technique for diagnosis and all-arthroscopic repair in TFCC Atzei class II and III lesions using a pre-tied suture device is a feasible and safe option with promising results.

**Supplementary Information:**

The online version contains supplementary material available at 10.1186/s13018-020-02046-1.

## Background

As the rise in knowledge of the wrist anatomy and injury mechanisms, triangular fibrocartilage complex (TFCC) injury becomes increasingly recognized as a rather common cause of ulnar-sided wrist pain [1–4]. Based on the clinical presentation and etiology, Palmer classification categorizes TFCC lesions into two types including traumatic tear (type I) and degenerative tear (type II), and has long been popularly used as a reference in treatment guide and outcome survey [5]. Palmer type I tear is further divided into four types depending on the location of tear; Palmer IB lesion refers to a peripheral tear and is most commonly encountered in patients with ulnar-sided wrist pain and disability. Recent evolution in arthroscopic techniques with better understanding in functional anatomy further clarifies pathophysiology and surgical policy in TFCC lesions.

The novel “iceberg” concept [6] has replaced the traditional “hammock” theory [7] to indicate the importance in repairing the submerged proximal component of TFCC and the need for renewing surgical approach. Refinement in fixation techniques has enabled arthroscopic fovea repair through either transosseous tunnel [8] or suture anchor insertion [9, 10]. The first purpose of this paper is to introduce a modified technique for differential diagnosis of Atzei class II and III lesions. The second purpose is to present an all-arthroscopic procedure using a pre-tied suture loop for TFCC Palmer IB lesions with proximal component involvement. Surgical technique is detailed, and preliminary outcome is reported.

## Methods

A modified all-arthroscopic technique for diagnosis and treatment of peripheral TFCC tear was proposed; 1-year preliminary outcomes in 12 patients were presented. The index surgery was started since early 2017 and has been performed in consecutive 35 patients (38 wrists) until paper submission. We obtained ethics committee approval from the Institutional Review Board for this study. Twelve patients with arthroscopically confirmed isolated peripheral TFCC tear involving the proximal component had more than 1 year follow-up, and clinical results were reported. There were 10 male and 2 female patients with a mean age of 33 years (range, 23 to 43). Right wrist was involved in 4 patients; three were in dominant hand. Left wrist was involved in 8 patients; three were in dominant hand. All recalled preceding injury history from minor strain to major accident and experienced ulnar-sided wrist pain for an average of 9.8 months (range, 3 to 24). Trauma mechanism included motorbike accident in 4 patients, work-related weight lifting in 3, sports injury in 2 (1 boxing aerobics and 1 bench press), and minor strain in 2.

### Surgical procedures

**Fig. 1:**
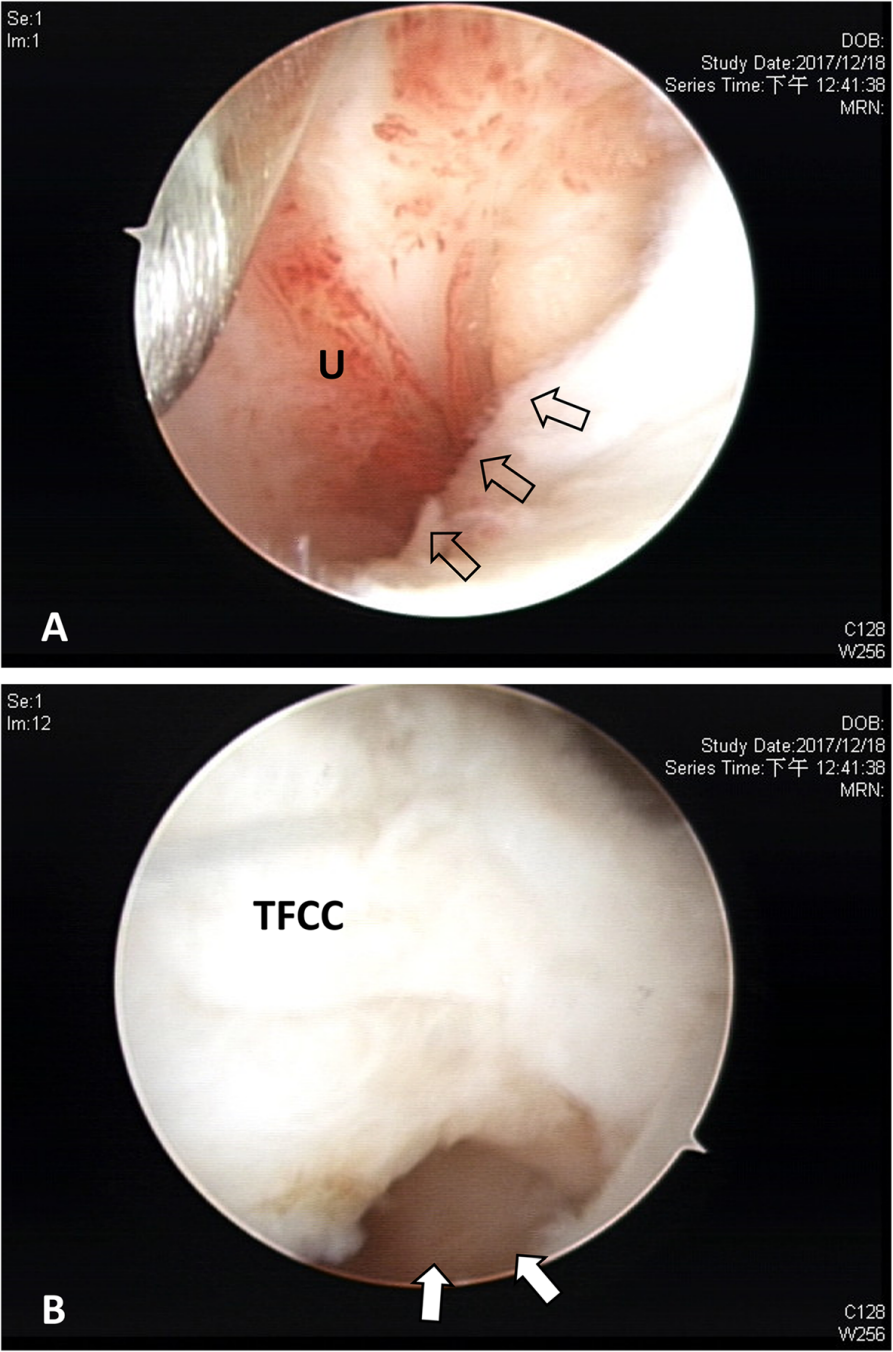
Thirty-five-year-old male patient, left ulnar-sided wrist pain for 3 months after motorbike accident. **a** Arthroscopic view from 3-4 portal shows detachment of TFCC periphery (hollow arrows) from ulnar joint capsule (U). **b** Arthroscopic view from 6R portal shows Atzei class II TFCC lesion with direct “visualization” of ulnar head (white arrows)


Arthroscopic technique was based on our previous publication in 2017 [11] while the routine exploration of dorsal sensory branch of ulnar nerve was not performed in this all-arthroscopic surgery. Under general anesthesia with the operated arm supported by a hand table, a pneumatic tourniquet was used with a mean pressure of 250 mmHg. By using a standard traction tower (ConMed, Largo, FL), finger traps were applied on index and ring fingers for longitudinal traction of 12 to 15 lb. Routine arthroscopic examination was started with a 30° 2.5-mm wrist arthroscope introduced through the standard 3-4 portal to expose a Palmar IB tear in the periphery of TFCC. Further diagnosis of the proximal component involvement was performed by shifting the arthroscope into the DRUJ portal where fovea tear and intact articular cartilage were confirmed. For discrimination between Atzei class II and III lesions, we performed three arthroscopic tests. With the arthroscope in the 3-4 portal with a probe inserted from the 6R portal to demonstrate hook test and trampoline test, the hook test was performed by inserting the probe into the prestyloid recess and applying a radially directed traction force to the periphery of TFCC [12, 13]. The trampoline test was used by probing the TFCC disc from 6R portal for visualizing TFCC compliance and detecting distal component tear. We then performed the 3rd test, which we called “visualization test,” on the distal component lesion by shifting the arthroscope to 6R portal and probing the TFCC periphery from 3-4 portal. A whole-layer avulsion of peripheral TFCC from ulnar capsule with direct visualization of ulnar head from 6R portal indicated tear of both distal and proximal components and referred to Atzei class II lesion (Fig. 1a and b). For Atzei class III lesion, partial thickness tear or scarring of the distal component could be directly visualized and thus clearly defined (Video 1).

**Additional file 1:** **Video 1.** 29 year-old male patient, left ulnar-sided wrist pain for 24 months after motorbike accident. Arthroscopic viewing from 6R portal shows triquetrum above and TFCC periphery below. A hook probe introduced from 3-4 portal showed partial thickness tear of the distal component; lower part of the distal component remains attached and blocks direct visualization of ulnar head.

**Fig. 2:**
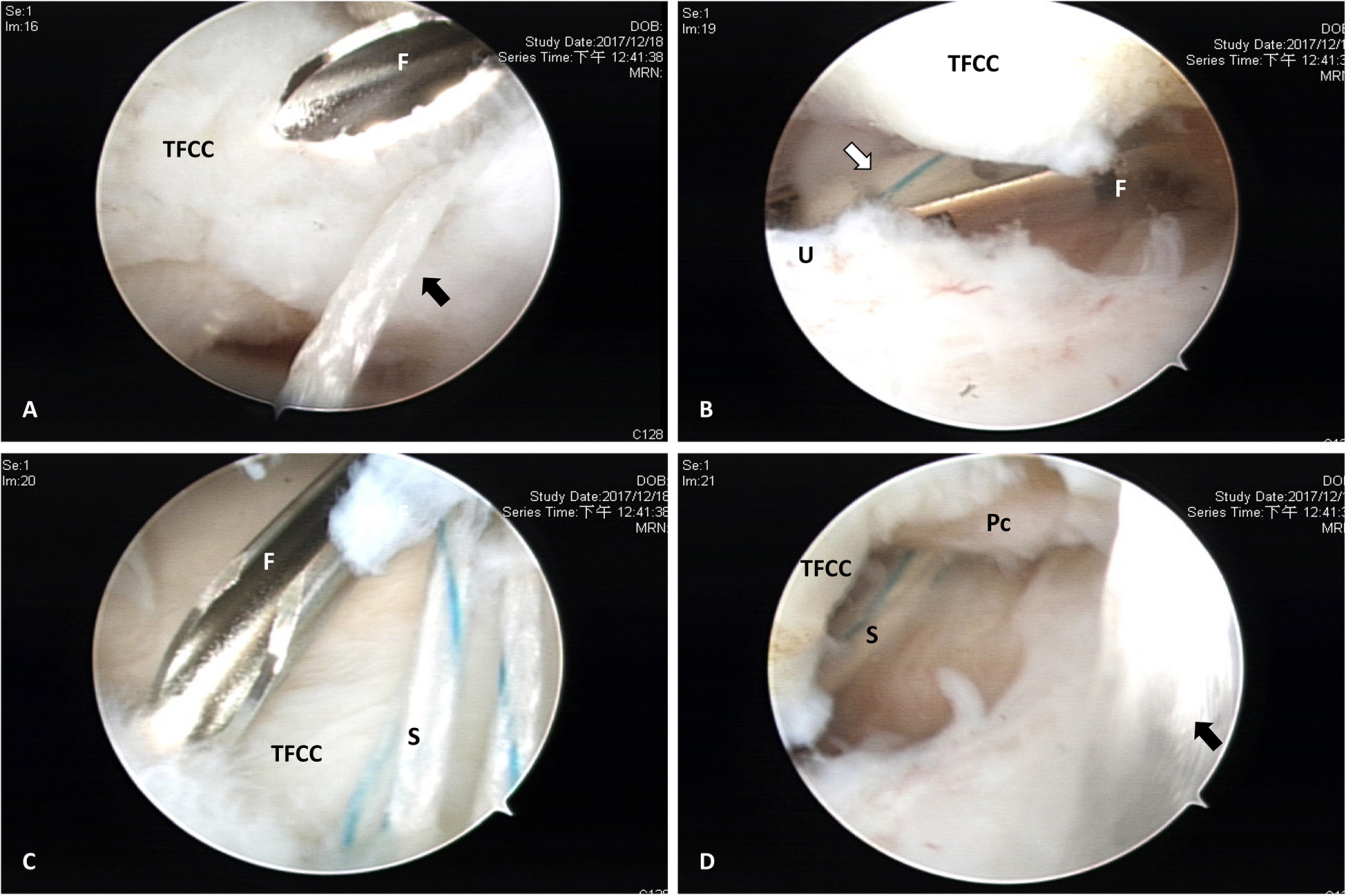
FasT-Fix suture device application for the patient in Fig. 1. Arthroscopic viewing from 6R portal. **a** Introduction of FasT-fix needle (F) from 3-4 portal for piercing TFCC by holding the TFCC in tension with ethibond suture (black arrow). **b** FasT-fix needle (F) penetrating and beneath TFCC for advancing the PEEK block (white arrow) to reach the ulnar joint capsule (U). **c** FasT-fix needle (F) from 3-4 portal about to pierce TFCC slightly volar to the first pierce with polyester suture (S) (black arrow). **d** Polyether suture (S) with twice piercing passes beneath TFCC and penetrate the proximal component (Pc) with ethibond suture (black arrow) kept in tension

**Fig. 3:**
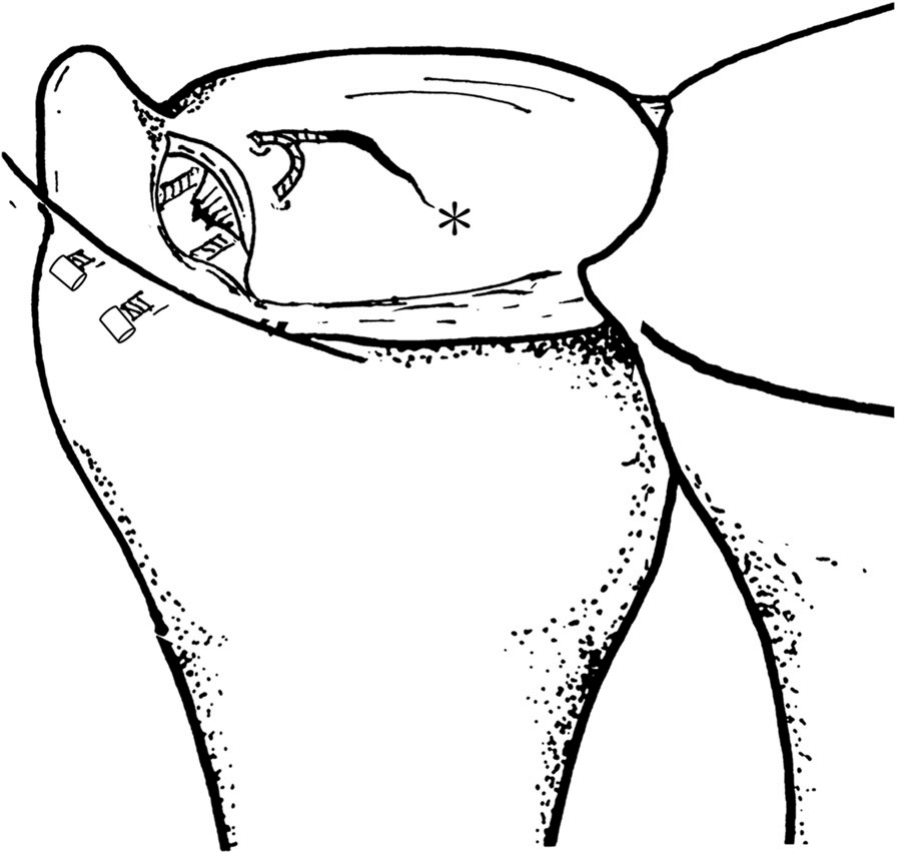
A suture loop was present at the periphery of TFCC; two suture limbs passed beneath the torn fiber and were distally locked with two poly-ether-ether-ketone (PEEK) blocks outside the ulnar joint capsule. Asterisk indicates a pre-tied polyester suture loop of the TFCC FasT-Fix device


After diagnostic arthroscopy and confirmation of TFCC lesion, a small joint motorized shaver was used for synovectomy and refreshment of the TFCC torn edge. Percutaneous 2-0 ethibond suture passing through #18 needle gauge was applied from 6U portal to pierce the distal component of TFCC and was used to check elasticity reparability of the distal component and provisionally approximate the tear gap (Video 2). Then, the arthroscope was shifted to 6R portal. TFCC FasT-Fix needle device (TFCC Fast-Fix, Smith & Nephew, Andover, MA) containing pre-tied polyester suture loop and two poly-ether-ether-ketone (PEEK) blocks were inserted from 3-4 portal and advanced to pierce through the peripheral TFCC on the volar side of the tear (Fig. 2) until passing ulnar joint capsule. The first PEEK block was pushed in through the device needle by releasing the trigger on the device handle to hold the ulnar joint capsule. This was followed by relocating the release trigger back to the original firing position. Then, the device needle was gently drawn back out of TFCC and re-advanced to pierce through the peripheral TFCC slightly dorsal to the first pierce until reaching the ulnar joint capsule again. The second PEEK block was inserted in the same way. Then, the device needle was completely withdrawn, and a pre-tied polyester suture loop was present in the periphery of TFCC with one suture limb outside the 3-4 portal (Fig. 3). The retention ethibond suture was held in proper tension with the distal radioulnar joint kept reduced in neutral rotation of the forearm by the assistant. The finger traction force was released, and a cutter/pusher instrument was inserted along the polyester suture limb through 3-4 portal. By gently retracting the suture limb and pushing the cutter/pusher to gradually tighten the pre-tied suture loop until the TFCC periphery was securely repositioned and held stably, the remaining polyester suture limb was then cut and removed. The arthroscope was then reintroduced through 3-4 portal for confirmation of repair quality by probing sutured TFCC from 6R portal (Video 3). The retention ethibond suture could be either removed or tightened as augmentation for large distal component tear in Atzei class II lesion. This was in the similar technique for outside-in suture repair with a small incision and meticulous dissection around the ethibond suture. Finally, the wound was closed with 4-O nylon suture.

**Additional file 3:** **Video 3.** The arthroscope is changed to 6R portal. By keeping the ethibond suture in tension, the polyester suture loop is tightened with a cutter-pusher. Then the arthroscope is shifted back to 3-4 portal with a hook probe from 6R portal to check TFCC stability after repair.

### Postoperative care

A short arm splint was applied with forearm supported on a sling and kept in neutral rotation for 6 weeks; the shoulder and elbow were allowed to move but wrist rotation was prohibited. Gentle wrist motion and rotation exercise started at 6 weeks after surgery with full activity resumed after 3 months. Then, regular follow-up was continued at 6 months and 1 year after surgery and every year thereafter.

## Results

Operation time from surgical time-out to completion of splinting averaged 87 min (range, 61 to 113). During arthroscopy from regular 3-4 portal, all wrists showed ulnar side peripheral tear of TFCC classified as Palmer IB lesion with positive hook and trampoline tests; DRUJ arthroscopy further confirmed proximal component tear in all cases. Among them, concomitant full thickness tear of the distal component was identified by visualization test from 6R portal and defined as Atzei class II lesion in four patients. In the remaining 8 patients, only partial thickness tear or scarring of the distal component was noted and defined as Atzei class III lesion. All 12 patients had a mean follow-up of 15 months (range, 12 to 24). Functional assessment based on Mayo modified wrist score (MMWS) improved from an average of 61.3 (range, 25 to 80) preoperatively to 90.4 (range, 70 to 100) at the latest follow-up. Mild residual pain on gym exercise or sports activity was noted in 3 patients; none was newly elicited symptom after surgery. All except one returned to work.

There was no major perioperative complication or infection. Two patients experienced transient paresthesia along the ulnodorsal side of the hand with gradual recovery subsequently. Both were cases of Atzei class II lesions and underwent augmented pullout suture repair of the distal component through 6U portal. All patients experienced tightness sensation or restriction on wrist motion especially rotation and radial deviation for the initially postoperative 6 months and did not cause limitation of motion at 1-year follow-up. Full activity was all resumed within 1 year after surgery.

## Discussion

The purpose of this study is to propose a modified arthroscopic technique for differential diagnosis and repair in isolated peripheral TFCC tear with proximal component involvement. Based on previous cadaver study in dynamic change of TFCC during rotation, the proximal component of TFCC is recognized as the true radioulnar ligament while the distal component functions like hammock suspension on the ulnar carpus [7]. Atzei et al. updates the concept in functional anatomy and proposed a treatment-oriented classification system with class II and class III lesions indicating proximal component involvement [13]. The hook test and trampoline test are commonly performed to discriminate type II versus type III lesions [6]. While positive hook test is a consistent indicator for fovea avulsion of the proximal component that could be confirmed through DRUJ arthroscopy, the trampoline test is an indirect sign for distal component tear by checking the TFCC compliance through compressive probing [14] instead of direct visualization in the integrity of the distal component. In our patients, trampoline test is all positive whereas only 4 exhibit complete tear in the distal component and is defined as Atzei class II lesion on arthroscopic viewing from 6R portal. In Atzei class II lesion, complete tear of both distal and proximal components allows direct visualization of ulnar head on inspecting TFCC periphery from 6R portal. We empirically call it positive visualization test. The other 8 patients, whose distal component is only in partial-thickness tear, are defined as Atzei class III lesion. Since all patients present fovea tear diagnosed with both positive hook test and DRUJ arthroscopy, it might be reasonable to lose a normal resilience response on probing for trampoline test. However, meticulous examination of the distal component from 6R portal is more straightforward to facilitate clear subtyping and allow proper surgical decision. Not only the thickness and size of the distal component tear was directly surveyed but also the whole procedure in passing TFCC FasT-Fix needle device could be meticulously monitored from 6R portal.

Both capsular and fovea repairs have been extensively adopted for surgical management of peripheral TFCC tear. Biomechanically, fovea repair is superior to capsule repair in terms of stiffness and maximal displacement of distal radioulnar joint (DRUJ) [15]. However, systematic reviews reveal comparable clinical outcomes among different surgical techniques since there is a lack of comparison studies regarding the area of TFCC injury and DRUJ instability [16]. In the index surgery, we implement an all-arthroscopic technique to re-approximate proximal component tear using a pre-tied suture device. There are two critical steps we believe worthy of reminding. Firstly, we use 6R portal to confirm an Atzei class II lesion through the “visualization” window in the peripheral tear, which in turn serves as a guide for FasT-fix device needle passing through the proximal component and ahead to ulnar joint capsule. Secondly, the pre-tied polyester suture loop is closed gradually by pulling the remaining limb to push down the TFCC periphery approaching ulnar fovea while keeping the retention ethibond suture in proper tension, forearm in neutral rotation, and DRUJ in reduction position. Similar to the currently available repair techniques, there is no direct evidence to document the healing status of proximal component. Nor do we have intention to repair the TFCC back to the original footprint or any isometric point. Instead, we may consider the index surgery to work like an internal bracing. The internal bracing technique has been currently adapted in treating ligament injury of elbow and acromioclavicular joints [17, 18]. By internal bracing of the torn ligaments, a suspension device was applied to stabilize the joint and facilitate tissue healing [19].

According to Atzei’s treatment algorithm [20], class II and III lesions are fovea-repaired through a DRUJ approach that is technically demanding and needs bony procedures with suture anchor or osseous tunnel. The all-arthroscopic technique using a pre-tied suture device in peripheral TFCC tear was first published by Yao et al. in 2007 [21]. Long-term report at a mean 7-year follow-up in Palmer IB lesion without gross DRUJ instability exhibits promising outcome [22]. We adapt this pre-tied suture device for use in the treatment of peripheral TFCC tear with proximal component involvement. With the arthroscope in the 6R portal, Palmer IB lesion can be clearly subcategorized. Under arthroscopic supervision, this pre-tied FasT-Fix device is introduced from 3-4 portal and advanced in the direction to re-approximate radial-displaced TFCC back to ulnar side. By pulling the retention suture in the 6U portal, the TFCC is held stable and kept in tension to facilitate accurate and sufficient purchase of the TFCC edge by the device needle. There exist several anatomical concerns regarding the safety of device needle application and PEEK block trajectory. Based on a cadaveric study for the FasT-Fix device [23], there is sufficient safe zone between the neurovascular structures and ulnar joint capsule where the needle tip reaches and PEEK blocks seat. Neither is any tendon injured. Biomechanical investigation shows a consistent failure mode with the suture cutting through the TFCC tissue. For prevention of cut-through complication, we recommend the use of smooth grasp forceps to check TFCC resilience and reparability in advance and then traction suture to facilitate needle passing. In our study, preliminary outcome for treatment of Atzei class II and III lesions is promising by an all-arthroscopic FasT-Fix suture repair, which is technically simpler and can serve as a feasible alternative to suture anchor or transosseous repair. The index surgery not only can be performed solely by skipping bony procedures, but also may serve as an adjunct whenever the proximal component or fovea condition is not allowed to achieve an optimal repair.

There are several drawbacks in our study. First of all, we do not explore the fovea area. Nor is postoperative image available to document the healing of TFCC periphery. In addition, there is lack of objective measurement to avoid over-tension of TFCC during tightening the pre-tied suture loop, which could be the cause of restricted motion in the early postoperative period. Finally, only a small sample size is recruited with the limitation of retrospective review.

## Conclusions

This study presents a modified technique for diagnosis and all-arthroscopic repair using a pre-tied suture device in Atzei class II and III TFCC lesions. The surgical procedures are simple and all through the portals for wrist arthroscopy without additional incisions. Preliminary outcomes are encouraging with limited complication.

## Supplementary Information

**Additional file 2:** **Video 2.** Percutaneous 2-0 ethibond suture passing through 6 U portal was used to check elasticity reparability of the distal component and provisionally approximate the tear gap.

## Data Availability

The datasets generated during the current study are available from the corresponding author on reasonable request.

## Abbreviations

TFCC: Triangular fibrocartilage complex
PEEK: Poly-ether-ether-ketone
DRUJ: Distal radioulnar joint
